# Supplementary material for: Myo-Inositol Moderates Glucose-Induced Effects on Human Placental 13C-Arachidonic Acid Metabolism
Source: Nutrients. 2022 Sep 26;14(19):3988. doi: 10.3390/nu14193988 (PMC9572372; doi:10.3390/nu14193988)
Supplement: Supplementary file 1 [file nutrients-14-03988-s001.zip › Suplementary Methods.pdf]

Supplementary Methods S1: Placental explant culture

Supplementary S1.1: Materials

Connaught Medical Research Laboratories (CMRL) media: GIBCO 1066-L Glutamine, Thermofisher, New York, USA. All media was sterilized by filtration though a 75-mm filter (Nalgene rapid flow™, 500 mL; Thermo Fisher Scientific). Bovine serum albumin: HI Clone fraction V, Culture grade, pH 7.00 lyophilized powder, GE Life Sciences, South Logan, Utah. Stable isotope labelled arachidonic acid: 1,2,3,4,5-^{13}C, 24 µmol/L, 95% pure, Cayman chemicals.

Supplementary S1.2: Placental explant production

Each placental biopsy was cut into small explants and explants washed with phosphate buffered saline (PBS). Five explants, with one explant coming from a separate placental biopsy, were added to each experimental well of 12-well plates (Nunclon™ Delta surface; Thermo Fisher Scientific) with each well containing 1.8 ml of media. All placenta processing was finished within 2 hours of delivery. Explants were incubated in a Thermo-scientific Foma Direct heat CO_2 Incubator, Hepa Class 100 Model 37 °C in a humidified atmosphere of 5% CO_2/air for 48 hours. Explants were removed from media and explants from triplicate wells combined and transferred into pre-weighed 2 ml Omni-tubes then washed with PBS. Excess PBS was removed after centrifugation and the explants frozen at -80°C.

Supplementary Methods S2: Lipid extraction

Frozen placental explants were freeze-dried, weighed, and then lysed using a bead-ruptor homogeniser and phosphate buffered saline (PBS, 1200 µl). Lysate (40 µl) was transferred to an Eppendorf containing 800 µL Butanol/Methanol (1:1) and 10 µl Internal standard mix (Supplementary Methods 3.2). All solvents were LCMS grade and were purchased from Merck except iso-propyl-alcohol which was purchased from Thermo-Fisher Scientific. Samples were

briefly vortexed, then sonicated for 30 minutes in an ice bath, then shaken for a further 30 minutes at 4 °C. Samples were then centrifuged at 13,000 rpm for 10 minutes, the supernatant transferred to a HPLC tube (*La-Pha-Pack, Germany*) and stored at -80 °C. Five quality control samples (BQC) and two blanks were extracted with every placenta. Quality control samples (BQC) were made from placenta lysate pooled from multiple participants, aliquoted and stored at -80 °C.

Supplementary Methods S3: LCMS Methodology

Supplementary S3.1: Sample analysis and LCMS methods

Lipid extracts (5 µl) were injected into an Agilent 6490 triple quadrupole (QQQ) liquid chromatography mass spectrometry (LC-MS/MS) instrument alongside a range of standards and analysed as described in Supplementary Methods 3.2. Samples were randomly analyzed with batch quality control samples (BQC) and blanks measured at regular intervals. ^{12}C-AA lipids were considered quantifiable if BQC percent standard deviation for that lipid was less than 25% and peak area was at least 10x that of the batch blank. ^{13}C-AA lipids were considered quantifiable if the peak co-eluted with a well quantified ^{12}C-AA counterpart, and if the peak area was at least 10x that of the batch blank and at least 3x that of placental explant lysate not incubated with ^{13}C-AA. ^{13}C-AA lipid transitions must also give peaks exactly co-eluting with their ^{12}C-AA counterpart, but with an increased mass of 5 Daltons (or multiples if more than one AA is incorporated). Only lipids with no missing data were included in the data set.

Chromatography was performed using a 2.1 x 100 mm 1.8 µm Zorbax Eclipse Plus C18 RRHD (Agilent Technologies) column at 60°C. and the following gradient: Mobile phase A: 50% water, 30% acetonitrile, 20% isopropanol, 10 mmol/L ammonium formate. Mobile phase B: 90% isopropanol, 9% acetonitrile, 1% water, 10 mmol/L ammonium formate. Start

(0.4 ml/min): 90% A, 0-2.7minutes: decrease to 55% A, 2.7-2.8 minutes: decrease to 47% A, 2.8-9 minutes: decrease to 35% A, 9-9.1 minutes: decrease to 11% A, 9.1-11 minutes: decrease to 8% A, 11-11.1 minutes: decrease to 0% A, 11.1 – 11.9 minutes: 0% A, 11.9 – 12 minutes: Increase to 90% A, 12 – 15 minutes: 90% A. The Agilent 6490 triple quadrupole was run with the following settings - Gas temperature: 150 °C. Gas flow: 17 L/min. Nebulizer: 20 psi. Sheath gas temperature: 200 °C. Sheath gas flow: 10 L/min. Positive capillary voltage: 3500 V, Negative capillary voltage: 3000 V. Positive nozzle voltage: 1000 V. Negative nozzle voltage: 1500 V. Positive high pressure RF (iFunnel): 100 V. Negative high pressure RF (iFunnel): 90 V. Positive low pressure RF (iFunnel): 100 V. Negative low pressure RF (iFunnel): 60 V. Fragmentor: 380. Polarity: positive.

Supplementary Methods S3.2 dMRM transition list and internal standard details

Lipid	RT	Transition	CE	IS
LPC 13:0 (IS) Avanti Polar lipids, 107 pmol per sample	1.65	454.3 -> 184.1	21	LPC 13:0
LPC_20.4	2.25	544.3 -> 184.1	21	LPC 13:0
LPC_20.4_13C	2.24	549.3 -> 184.1	21	LPC 13:0
PC 13:0 13:0 (IS), Avanti Polar lipids, 72.1 pmol per sample	4.73	650.5 -> 184.1	21	PC 13:0 13:0
PC 40:8	6.04	830.6 -> 184.1	21	PC 13:0 13:0
PC 40:8_13C	6.06	835.6 -> 184.1	21	PC 13:0 13:0
PE 17:0/17:0 (IS), Avanti Polar lipids. 100.7 pmol per sample	8.80	720.6 -> 579.5	17	PE 17:0/17:0
PE 38:4	8.11	768.6 -> 627.6	17	PE 17:0/17:0
PE 38:4_13C	8.11	773.6 -> 632.6	17	PE 17:0/17:0
PE 38:5	7.15	766.5 -> 625.5	17	PE 17:0/17:0
PE 38:5_13C	7.15	771.5 -> 630.5	17	PE 17:0/17:0
PE(P-36:4)	7.53	724.5 -> 361.3	17	PE 17:0/17:0
PE(P-36:4)_13C	7.53	729.5 -> 366.3	17	PE 17:0/17:0
PE(P-38:4)	8.75	752.6 -> 361.4	17	PE 17:0/17:0
PE(P-38:4)_13C	8.75	757.6 -> 366.4	17	PE 17:0/17:0
PI 38:4	6.16	904.6 -> 627.6	17	PE 17:0/17:0
PI 38:4_13C	6.16	909.6 -> 632.6	17	PE 17:0/17:0
TG_50:4	11.08	844.8 -> 523.5	21	TG_51:0
TG_50:4_13C	11.08	849.8 -> 523.5	21	TG_51:0
TG_51:0_(17:0 17:0 17:0) (IS) Sigma Aldrich, 85.1 pmol per sample	11.84	866.8 -> 579.5	21	TG_51:0

TG_52:5	11.10	870.8 -> 549.5	21	TG_51:0
TG_52:5_13C	11.10	875.8 -> 549.5	21	TG_51:0
TG_54:4	11.49	900.9 -> 579.6	21	TG_51:0
TG_54:4_13C	11.49	905.9 -> 579.6	21	TG_51:0
TG_54:5	11.29	898.9 -> 577.6	21	TG_51:0
TG_54:5_13C	11.29	903.9 -> 577.6	21	TG_51:0
TG_54:6	11.13	896.8 -> 575.5	21	TG_51:0
TG_54:6_13C	11.13	901.8 -> 575.5	21	TG_51:0
TG_54:7	10.99	894.8 -> 573.5	21	TG_51:0
TG_54:7_13C	10.99	899.8 -> 573.5	21	TG_51:0
TG_56:6	11.33	924.9 -> 603.6	21	TG_51:0
TG_56:6_13C	11.33	929.9 -> 603.6	21	TG_51:0
TG_56:7	11.17	922.8 -> 601.5	21	TG_51:0
TG_56:7_13C	11.16	927.8 -> 601.5	21	TG_51:0
TG_56:8	11.07	920.9 -> 599.6	21	TG_51:0
TG_56:8_13C	11.08	925.9 -> 599.6	21	TG_51:0
TG_58:8	11.28	948.8 -> 627.5	21	TG_51:0
TG_58:8_13C	11.28	953.8 -> 627.5	21	TG_51:0
TG_58:9	11.10	946.8 -> 625.5	21	TG_51:0
TG_58:9_13C	11.09	951.8 -> 625.5	21	TG_51:0

Supplementary methods S4: Data analysis and statistics

LC-MS/MS data was analysed using Mass Hunter QQQ Quantitative Analysis Version 8 and peak area quantified by integration. Data was analyzed in R version 4.1.1 (Kick Things) using the tidyverse, tidymodels dplyr, purrr, broom, readr, moderndive, rstatix, dlookr packages. Graphs were made using the ggplot2, ggpubr, ggrepel, ggforce, ggthemes and viridis packages. Only lipids with no missing values below the limits of quantification were included in the dataset so no assumptions were made for any lipids analyzed. A few glucose or myo-inositol experiments contained incomplete datasets due to technical issues in processing a few samples. These included 5 mM glucose and 60 µM MI (2 samples), 10 µM glucose and 0.3 µM MI (2 samples), 10 µM glucose and 60 µM MI (1 sample), 17 µM glucose and 0.3 µM MI (1 sample) and 17 µM glucose and 60 µM MI (1 sample). Peak areas were normalized against their corresponding lipid class internal standard to give

concentration expressed as pmol/ml. Dried placental mass was used to calculate the lipid amount expressed as pmol/ dry mg placenta using the following calculations:

Amount of lipid in sample= Peak area lipid/ Peak area internal standard*amount of internal standard in sample

Proportion of lysate used to prepare sample = 40 µl / total lysate (1200 µl) = 30

Amount of lipid in lysate = Amount of lipid in sample*30

Amount of lipid per mg dry placenta = Amount of lipid in lysate/ mass dry placenta used to prepare lysate

Supplementary methods S5: Placental biopsy cohort

Supplementary S5.1

Placentas were obtained from 26 non-GDM and 24 GDM singleton pregnancies delivered by elective caesarean section at term at the National University Hospital, Singapore between 2017 – 2018. GDM was diagnosed using the WHO 2013 criteria; i.e. fasting glucose ≥ 5.1 mmol/l, 1-hour ≥ 10.0 mmol/l, 2 hour ≥ 8.5 mmol/l as measured by a 75 g oral glucose tolerance test (OGTT) at mid-gestation. Pregnancies were without other complications. Women were recruited with informed written consent. Ethical approval was obtained from the National Healthcare Group Domain Specific Review Board (2016/00183). Biopsies of the villous placental tissue were frozen in liquid nitrogen within 10 minutes of delivery. Five biopsies from each placenta were then crushed in liquid nitrogen to create a representative sample, from which 250 mg was freeze-dried, weighed and lysed in 1 ml phosphate buffered saline using an Omni bead rupter. Lipids were extracted from the placental lysate with butanol and methanol (1:1, containing internal standards) and stored at -80 ºC until analysis. Lipid extracts were injected into an Agilent 6490 triple quadrupole LC-MS/MS instrument (Agilent) and analyzed using a targeted dMRM method. Total placental inositol was extracted as previously described [33]. The Megazyme® kit (Dublin, Ireland), an enzymatic assay for inositol quantification was used following manufacturer's guidelines with modifications [80].

Supplementary S5.2: Clinical characteristics of participants in the study

	Non GDM (N=26)	GDM (N=24)	BMI<25 (N=25)	BMI>25 (N=25)
Maternal Age (yrs)	32.7 (4.1)	33 (3.5)	32.9 (3.9	32.8 (3.7)
Maternal BMI (kg/m²)	23.5 (3.1)	28 (5.8)	21.8 (2.0)	29.6 (4.1)
Maternal Fasting Glycemia[#] (mmol/L)	4.3 (0.3)	4.9 (0.3)	4.4 (0.3)	4.7 (0.4)
Maternal 2 hr Glycemia[#] (mmol/L)	5.8 (0.9)	8.8 (1.5)	6.6 (1.8)	8.0 (1.9)
Gestation at Delivery (Days)	270.1 (3.7)	269.3 (3.9)	269.6 (3.5)	269.8 (4.0)
Fetal Sex – Male (%)	73.1	45.8	60	60
Birth weight (g)	3238 (297)	3389 (282)	3280 (276)	3341 (320)

Values represent mean (±SD) or N (%). [#] Glucose results from a mid-gestation 75g oral glucose tolerance test. Abbreviations: BMI Kg/m^2, Body Mass Index; GDM, Gestational diabetes mellitus.
