# Supplementary material for: Myo-Inositol Moderates Glucose-Induced Effects on Human Placental 13C-Arachidonic Acid Metabolism
Source: Nutrients. 2022 Sep 26;14(19):3988. doi: 10.3390/nu14193988 (PMC9572372; doi:10.3390/nu14193988)
Supplement: Supplementary file 1 [file nutrients-14-03988-s001.zip › Supplementary Figures.pdf]

Alterations in ^{12}C-AA lipids

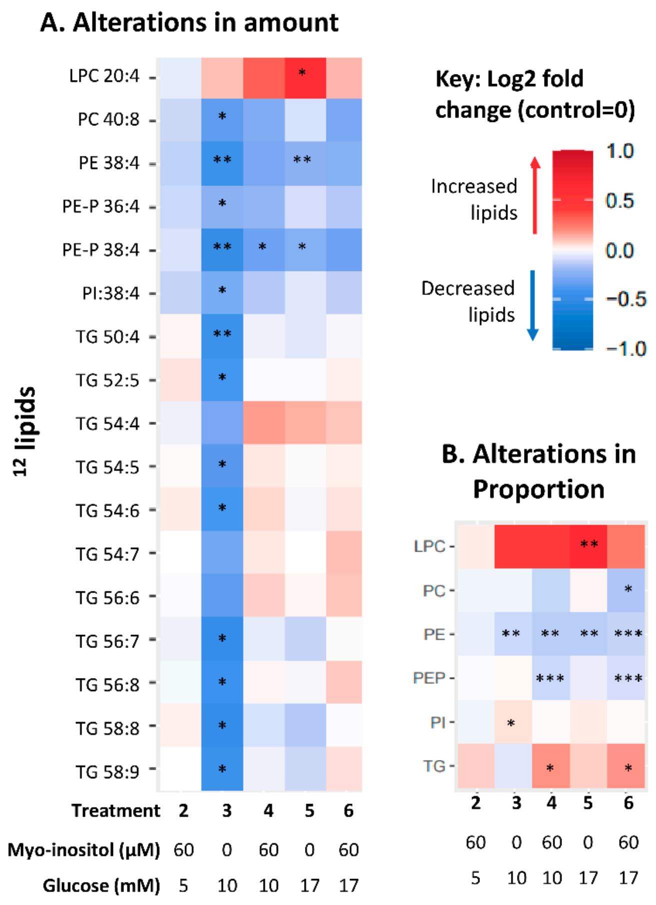

Supplementary Figure S1. **Heat map illustrating alterations in ^{12}C-AA lipids in placental explants in response to glucose and myo-inositol treatment.** Color indicates the relative change (Log2-fold change) in AA lipids, in placental explants treated with myoinositol (60 µM) and/or glucose (10 or 17 mM) compared with controls from the same placenta treated with no additional glucose (5 mM) or myoinositol (0.3 µM). A: alterations in amount. B: Alterations in the proportion of each ^{12}C-AA lipid class relative to total quantified ^{12}C-AA-lipids. Asterisks indicate significant differences by one sample T test compared to control (test value=0) after Benjamini-Hochberg correction for multiple comparisons *p<0.05, **p<0.01, ***p<0.001.

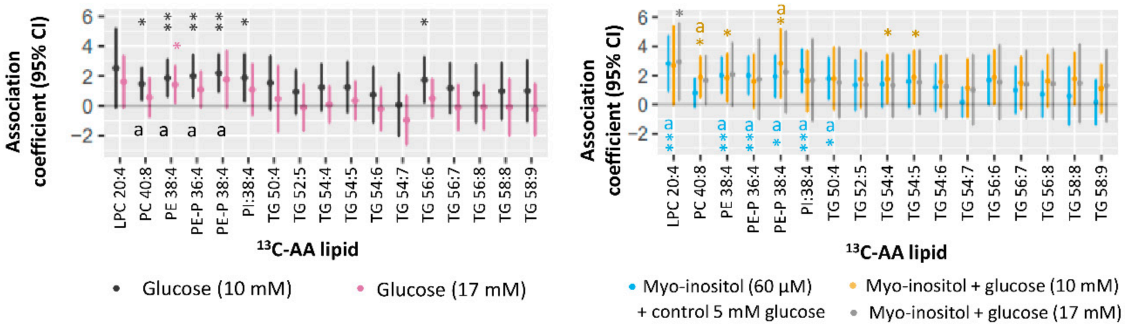

Supplementary Figure S2. Forest plots showing positive associations of fasting glycemia with alterations in ^{13}C lipids (log2-fold change) induced by glucose, myo-inositol and combination glucose myo-inositol. Treatment induced alterations represent the relative amount of ^{13}C-AA lipid in treated placental explants treated compared to control explants from the same placenta. Positive log2-fold-change values indicate an increase in ^{13}C-AA lipids compared to the control, whilst negative values indicate a decrease. Linear regression was run with treatment response as the outcome and fasting glycemia as the exposure variable. Benjamini-Hochberg (BH) method was used to correct for multiple testing. Forest plot show association coefficients and 95% confidence intervals. Asterisks indicate significant associations *p<0.05, **p<0.01.

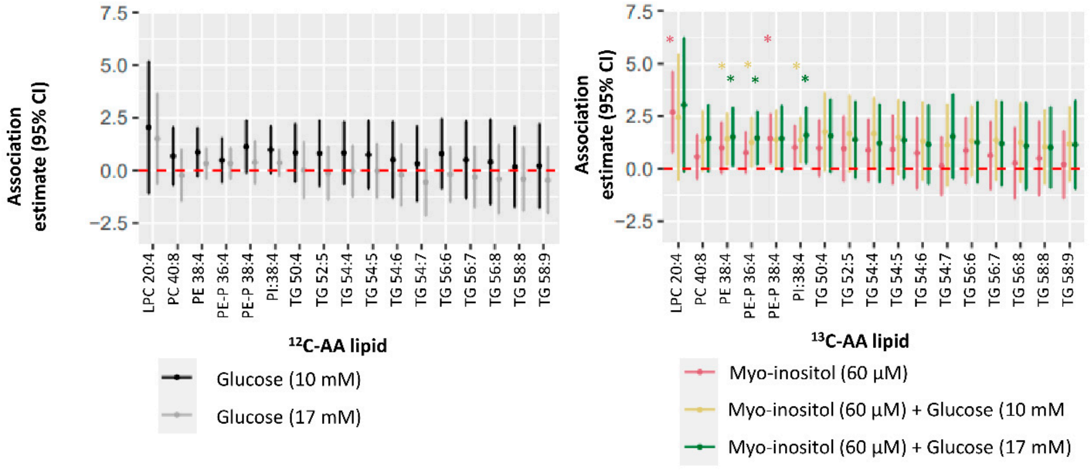

Supplementary Figure S3. Forest plots showing positive associations of fasting glycemia with alterations in ^{12}C lipids (log2-fold change) induced by glucose, myo-inositol and combination glucose myo-inositol. Treatment induced alterations represents the relative amount of ^{12}C-AA lipid in treated placental explants compared to control explants from the same placenta. Positive log2-fold-change values indicate an increase in ^{13}C-AA lipids compared to the control, whilst negative values indicate a decrease. Linear regression was run with treatment response as the outcome and fasting glycemia as the exposure variable. Benjamini-Hochberg (BH) method was used to correct for multiple testing. Forest plot showing association coefficients and 95% confidence intervals. Asterisks indicate significant associations *p<0.05, **p<0.01.

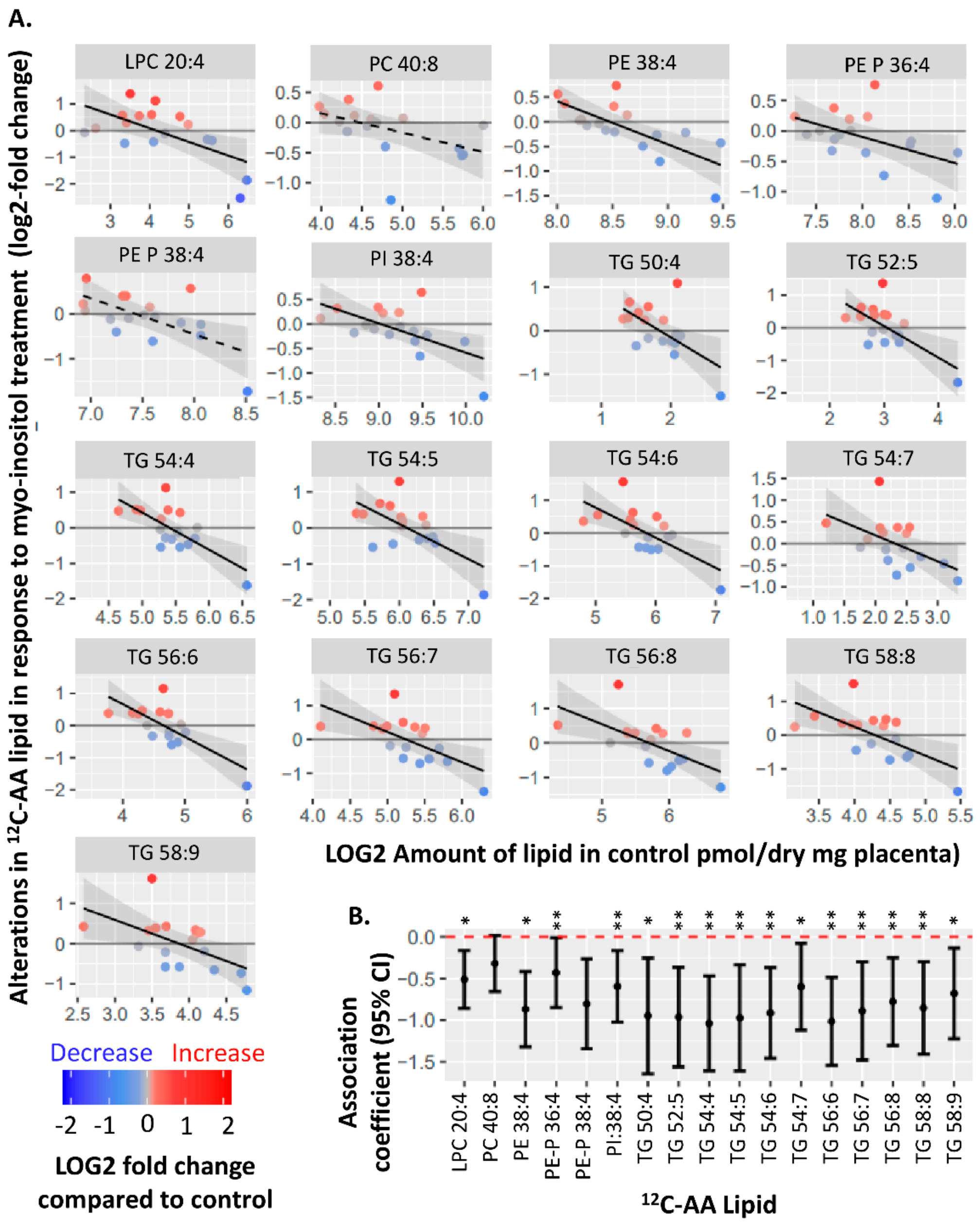

Supplementary Figure S4. Negative associations between amount of each ^{12}C-AA lipid in the control and alterations in ^{12}C-AA lipid (log2-fold change) in response to myo-inositol treatment. Myo-inositol induced alterations represents the relative amount of ^{12}C-AA lipid in placental explants treated with myo-inositol compared to control explants from the same placenta. Positive log2-fold-change values indicate an increase in ^{13}C-AA lipids compared to the control, whilst negative values indicate a decrease. Linear regression was run with myo-inositol response as the outcome and fasting glycemia as the exposure variable. Benjamini-Hochberg (BH) method was used to correct for multiple testing. A) Solid lines show significant associations while dashed lines show non-significant trends, shaded areas show 95% confidence intervals. B) Forest plot show association coefficients and 95% confidence intervals. Asterisks indicate significant associations *p<0.05, **p<0.01.
